# Perceived Threat of COVID‐19 and Vaccination Coverage Among Nurses: A Multicentre Cross‐Sectional Study

**DOI:** 10.1002/nop2.70469

**Published:** 2026-03-09

**Authors:** Marina Moreno‐Martínez, Maria Feijoo‐Cid, Josep Maria Manresa‐Dominguez, Antonia Arreciado Marañón, Esperanza Zuriguel‐Pérez, Sandra Ponce‐Ruíz, Ramón Sebastián Torrente‐Jimenez, Maria Isabel Fernández‐Cano

**Affiliations:** ^1^ Department of Medicine, Faculty of Medicine Universitat Autònoma de Barcelona Bellaterra Spain; ^2^ Súria Primary Care Centre Catalan Institute of Health Barcelona Spain; ^3^ Faculty of Health Sciences University of Manresa Barcelona Spain; ^4^ Department of Nursing, Faculty of Medicine Universitat Autònoma de Barcelona Bellaterra Spain; ^5^ Grup de REcerca Multidisciplinar en SAlut i Societat (GREMSAS), (2021SGR1484) Mataró Spain; ^6^ Fundació Institut Universitari Per a la Recerca a L’atenció Primària de Salut Jordi Gol i Gurina (IDIAPJGol‐UAB) “Unitat de Suport a la Recerca Metropolitana Nord” Mataró Spain; ^7^ Grupo de Investigación Multidisciplinario de Enfermería Vall D’hebron Instituto de Investigación (VHIR) Barcelona Spain; ^8^ Hospital de Traumatología, Rehabilitación y Quemados Hospital Universitario Vall D’hebron Barcelona España; ^9^ Consejería de Educación, Universidades, Cultura y Deportes de Canarias Las Palmas de Gran Canaria Gran Canaria Spain

**Keywords:** attitudes, COVID‐19, nurses, threat perception, vaccine hesitancy

## Abstract

**Aim:**

Analyse the perceived threat of COVID‐19, vaccination coverage and associated factors among nurses in Barcelona (Spain) in 2021 after the start of the vaccination campaign.

**Design:**

Multicentre cross‐sectional study using an anonymous online questionnaire accessible from 26 February to 31 May 2021. Cherries guidelines were followed.

**Methods:**

Three hundered and eighty nine hospital and primary care nurses participated using an anonymous online questionnaire accessible from 26 February to 31 May 2021. The questionnaire included ad‐hoc questions and the validated Questionnaire on the Perceived Threat of COVID‐19. Univariate and bivariate analyses were performed based on the type of variable.

**Results:**

Perceived threat was higher among female nurses, the 35–49 age group and the hospital setting. Being a woman, living with a dependent and believing oneself vulnerable to falling ill were all associated with this perceived threat. Vaccination coverage was high in general and even higher among women despite having higher scores for anxiety from thinking about vaccination. Most nurses trusted the efficacy and safety of the vaccine.

**Patient or Public Contribution:**

No patient or public contribution.

**Conclusions:**

Despite having already been vaccinated, the nurses presented a significant perceived threat, with women with dependents feeling the most threatened. The attitude of the nurses towards vaccination was good since they trusted the efficacy and safety of the vaccine.

**Implications for the Profession:**

Nurses are frontline healthcare workers (HCWs) in any pandemic, so strategies are needed to (1) help them manage perceived threat and (2) debunk false beliefs that prevent vaccination. Training might have been offered on how to manage the physical and mental overload of the pandemic and how to improve the consultation of scientific resources to obtain accurate, evidence‐based information. Public confidence should be increased through communication and education, such as vaccination reminders and debunking fake news—social networks are a major channel among young people and useful for sharing scientific information.

**Reporting Method:**

This study adheres to the EQUATOR Checklist for Reporting Results of Internet E‐ Surveys (CHERRIES).

## 1 Introduction

The COVID‐19 pandemic has been a global public health problem. There were six epidemic waves in the first 2 years (2020–2022). In the first wave, lasting until April 2020, the rate of HCWs affected by COVID‐19 internationally was 10.1%, with an incidence of severe disease of 9.9% and a mortality rate of 0.3% (Sahu et al. 2020). Nurses were the HCWs most affected, representing 48% of cases (Gomez‐Ochoa et al. 2020). Healthcare systems across the globe were forced to quickly reorganize to handle this exceptional situation (Barello et al. 2020). As part of the fight against COVID‐19, research was conducted to identify effective drugs to prevent infection and develop vaccines, several of which were eventually authorized by the European Medicines Agency (Comité Asesor de Vacunas 2021). Initially, HCWs were more hesitant to be vaccinated against COVID‐19 than seasonal influenza (Dror et al. 2020). Before being commercialized, the rate of acceptance of COVID‐19 vaccines was 78% among physicians and 61% among nurses, lower even than that of the general population (75%) (Dror et al. 2020). In early 2022, 49.2% of the world population was fully vaccinated against COVID‐19 (World Health Organization 2022), which has led to a major reduction in the mortality of infected people. The available data show that 73.6% of those fully vaccinated account for only 19.4% of deaths from COVID‐19, while 20.5% of unvaccinated people represent 75.3% of deaths (Washington State Department of Health 2022).

## 2 Background

Perceived threat has been considered one of the determining factors in the decision of HCWs to get vaccinated against COVID‐19. Those who did not perceive COVID‐19 as a threat were less willing to get vaccinated (Dzieciolowska et al. 2021). Perceived threat is related to an individual's interpretation of the experience of becoming ill: in other words, how they perceive the disease, its aetiology, treatment, evolution and cure. The Common‐Sense Model of Self‐Regulation helps explain this perceived health threat. The model focuses on the generation of a mental representation of the threat and associated emotions to promote coping strategies (Pérez‐Fuentes et al. 2020). Other available evidence does not use the term “perceived threat”, but rather speaks of perceived risk, such that a high self‐perceived risk of severe COVID‐19 infection is considered a positive predictor for vaccination. On the other hand, negative predictors for its acceptance include factors such as being a nurse, having children (Dror et al. 2020), questioning the safety of the vaccine, including concerns about the short‐ and long‐term consequences and the belief that the risk is greater than the benefit. There are also doubts about its efficacy, such as the belief that the vaccine does not offer protection or that it is short‐lived. Other factors include the swift development of the vaccine, a lack of information, having had reactions to previous vaccines and a fear of needles (Dzieciolowska et al. 2021).

Past studies have shown that nurses have been hesitant to get vaccinated against other diseases such as influenza (On et al. 2021).

## 3 The Study

### 3.1 Aim

This study aims to analyze the perceived threat of COVID‐19, vaccination coverage and associated factors among nurses in Barcelona (Spain) in 2021 after the start of the vaccination campaign.

## 4 Methods

### 4.1 Design and Setting

Multicentre cross‐sectional study using an online questionnaire. We invited 2905 nurses from Vall d’Hebron University Hospital in Barcelona, Spain, to respond. This is one of the largest tertiary hospitals in Europe, with more than 1000 beds. We also invited 1300 primary care (PC) nurses from the North Metropolitan health district of Barcelona to participate. The CHERRIES guidelines for reporting research results from online questionnaires were followed (López‐Rodríguez 2019).

### 4.2 Sample Size

We calculated that a minimum sample of 214 nurses was needed, with an alpha risk of 0.05 and a margin of error of ±0.3 units. The standard deviation of the population mean of the expected perceived threat was 2 units, and an additional 20% was estimated for the replacement of study dropouts.

### 4.3 Sampling and Recruitment

Snowball sampling was used. An invitation email was sent to all nurses on the hospital and PC centre distribution list. It explained the objectives of the study and had a link to the questionnaire. Participation was voluntary and did not involve economic compensation.

### 4.4 Data Collection

The questionnaire was first distributed to HCWs 2 months after the start of the vaccination campaign and was accessible from 26 February to 31 May 2021. Additionally, two reminders were sent seven and 15 days after the initial invitation. The duration of the data collection period ran from the end of the third wave to the end of the fourth wave in Spain. The cumulative incidence was around 232.6 cases per 100,000 inhabitants (Centro de Coordinación de Alertas y Emergencias Sanitarias 2021), with 80% of HCWs vaccinated in March (Cofares 2022) and 7.2% of the general population vaccinated in April (de Sanidad 2021).

### 4.5 Instruments and Variables

A self‐administered questionnaire with 26 closed questions in a single page was used via the Research Electronic Data Capture platform (REDCap) (Harris et al. 2019), a secure web app that complies with international standards. All the questions had to be answered in order to complete the survey; they were the same for everyone, without randomization or conditionally displayed items. All responses were automatically saved into the website's database. The questionnaire included ad‐hoc questions to explore: (1) sociodemographic data and experience with COVID‐19, (2) the Questionnaire on the Perceived Threat of COVID‐19, (3) vaccination coverage and (4) attitudes towards vaccination. The Questionnaire on the Perceived Threat of COVID‐19 consists of five items responded to on a Likert scale of 0–10, where higher scores indicate a greater perceived threat of COVID‐19. The internal consistency of the questionnaire is α = 0.66 and it was validated for the Spanish population (Pérez‐Fuentes et al. 2020). The questions related to attitudes towards vaccination were extracted from the report on nurses’ perception of influenza vaccination by the Nurses Professional Association of Spain (Organización Colegial de Enfermería 2020). The following variables were adapted: motivation for vaccination, perceived efficacy and safety of the vaccine, ethical responsibilities related to vaccination and perception of the severity of COVID‐19 infection. The ad‐hoc questions for the remaining variables were designed based on a literature review of related published studies, adapting them to the specific circumstances (Table SS1). The final questionnaire was reviewed by expert nurses with professional experience in COVID‐19 and vaccination. It was piloted with 10 nurses, whose responses were not included in the analysis. The response time range varied between 15 and 20 min.

### 4.6 Analysis

A univariate descriptive analysis was performed, estimating the absolute and relative frequencies of the qualitative variables and the mean and standard deviation of the quantitative variables. A bivariate descriptive analysis was performed using the Student's *t*‐test, ANOVA and Chi‐squared test to determine any potential association between perceived threat among HCWs and the sociodemographic and work variables, COVID‐19 experience and attitude towards vaccination. The analysis was performed using the computer software SPSS version 26.0.

### 4.7 Ethical Considerations

The study was approved by the two Clinical Research Ethics Committee. Confidentiality and anonymity were ensured during data collection and management. Participants granted their consent for their data to be analysed.

## 5 Results

A total of 389 nurses answered the questionnaire, of which 261 (67.1%) were nurses working in hospitals and 115 (29.6%) in PC. Based on the total number of workers on the mailing list, the overall response rate was 8.9% and 9.0% for hospital nurses and 8.8% for PC nurses.

### 5.1 Sociodemographic Characteristics and Experience With COVID‐19

A total of 336 participants (86.4%) were women and the mean age was 41.8 years (SD: 12.0). 223 (57.3%) nurses worked in the public sector with a temporary contract, and 42 (10.8%) worked at more than one centre. A total of 101 (26.2%) nurses had a positive polymerase chain reaction (PCR) test for SARS‐COV‐2, and 297 (76.3%) had a family member or friend infected with COVID‐19 (Table 1).

**TABLE 1:** Relative frequencies of the sociodemographic variables and personal experience with COVID‐19.

	Work setting		TOTAL (%)
	Hospital *n* = 261	Primary care *n* = 115	
Sex			
Male	13.0	14.8	13.6
Female	87.0	85.2	86.4
Age			
21–34 years	37.4***	20.2	32.3
35–49 years	31.1	45.6***	35.7
≥ 50 years	31.5	34.2***	32.0
Dependents	31.4	51.3***	37.8
Working at more than one health centre	8.9	14.8	10.8
Contract type			
Permanent	42.9	43.5	42.7
Temporary	57.1	56.5	57.3
Professional experience as nurse			
0 to < 2 years	8.0	5.2	7.2
2 to < 5 years	14.6	7.8	12.3
5 to < 10 years	15.3	9.6	14.1
≥ 10 years	62.1	77.4	66.3
Front‐line work	72.0	93.0***	78.7
COVID‐19 diagnostic tests performed			
PCR	97.7	96.5	97.2
RAT	16.9	60.9***	29.8
Specific antibody test	49.0	51.3	50.1
Non‐specific antibody test	10.0	15.7	11.8
Reason for COVID‐19 diagnostic tests performed			
Symptoms	35.2	42.6	37.8
Close contact	29.7	23.5	27.9
Screening	41.4	40.0	41.3
Positive PCR	27.1	24.3	26.2
Quarantine compliance	44.1	52.2	46.8
Positive antibody test	26.4	29.6	27.2
Family member/friend affected by COVID‐19	77.8	72.2	76.3

*** *p* < 0.001.

### 5.2 Perceived Threat

The mean overall score of perceived threat was 29.1 (95% CI 28.4–29.9) out of a maximum score of 50 points. Scores were higher among women, participants in the 35–49 age group and hospital nurses. Male nurses and PC nurses more frequently reported believing that the disease would have a long duration (Table 2).

**TABLE 2:** Perceived threat of COVID‐19 by sex, age and work setting.

	TOTAL $\left(\bar{X}\pm \mathrm{SD}\right)$	Sex		Age			Work setting	
		Male *n* = 53	Women *n* = 336	16–34 *n* = 123	35–49 *n* = 136	≥ 50 *n* = 122	Hospital *n* = 261	Primary care *n* = 115
How much does being infected by COVID‐19 affect your life? (0–10)	6.9 ± 2.1	6.5 ± 2.5	7.0 ± 2.0	7.2 ± 1.9	7.0 ± 2.1	6.6 ± 2.2	6.9 ± 2.1	6.8 ± 2.2
How long do you think the COVID‐19 infection will last? (0–10)	7.3 ± 1.7	7.6 ± 2.0	7.3 ± 1.7	7.2 ± 1.6	7.5 ± 1.8	7.3 ± 1.8	7.3 ± 1.7	7.5 ± 1.8
To what extent do you feel COVID‐19 symptoms? (0–10)	1.9 ± 2.5	1.5 ± 2.4	2.0 ± 2.5	2.2 ± 2.5	1.8 ± 2.5	1.7 ± 2.5	1.9 ± 2.5	1.8 ± 2.5
To what extent are you concerned about being infected by COVID‐19? (0–10)	6.4 ± 2.4	5.0 ± 2.9	6.7 ± 2.2***	6.2 ± 2.3	6.6 ± 2.5	6.5 ± 2.4	6.5 ± 2.4	6.2 ± 2.4
To what extent does being infected by COVID‐19 affect you emotionally? (0–10)	6.625	6.0 ± 3.0	6.7 ± 2.5*	6.7 ± 2.5	6.9 ± 2.3	6.1 ± 2.7	6.7 ± 2.5	6.2 ± 2.5
Total score:	29.1 ± 7.3	26.5 ± 8.6	29.6 ± 6.9**	29.5 ± 6.6	29.9 ± 7.1	28.1 ± 7.9	29.3 ± 7.2	28.6 ± 7.0

* *p* < 0.05.

** *p* < 0.01.

*** *p* < 0.001.

There was a significant association between perceived threat and being a woman, in which the score was 29.6 (SD: 6.9), as compared to 26.5 (SD: 8.6) in men (*p* < 0.01). Living with dependents was also associated with perceived threat, with a mean difference of 1.7 points compared to those who did not have dependents (*p* < 0.05). Regarding experience with COVID‐19, having had a positive COVID‐19 antibody test was associated with a mean perceived threat of 30 (SD: 8.4) while a negative antibody test was associated with a mean perceived threat of 28.8 (SD:6.8) (*p* < 0.05). Feeling vulnerable to falling ill with COVID‐19 was directly associated (*p* < 0.001) with perceived threat while thinking that most cases of COVID‐19 infection do not present severe symptoms was indirectly associated (*p* < 0.01) with perceived threat. Regarding vaccination, the level of anxiety related to COVID‐19 vaccination (*p* < 0.001), working in areas of risk as the motivation to get vaccinated (*p* < 0.05) and health problems of the HCWs as the motivation to get vaccinated (*p* < 0.05) were all directly related to perceived threat, while having been vaccinated was not related.

### 5.3 Vaccination Coverage

A total of 337 (86.6%) nurses were vaccinated against COVID‐19. The overall mean score of anxiety related to vaccination was 2.8 (SD 3.1) points and was higher in women than in men by 0.9 points (*p* < 0.05) (Table 3).

**TABLE 3:** Percentage of COVID‐19 vaccination coverage, reasons for vaccination, perception of vaccine efficacy and safety and perception of the severity of COVID‐19 infection.

	TOTAL (%)	Sex		Age			Work setting	
		Male *n* = 53	Women *n* = 336	21–34 *n* = 123	35–49 *n* = 136	≥ 50 *n* = 122	Hospital *n* = 261	Primary care *n* = 115
**Vaccination coverage**								
I have been vaccinated against COVID‐19	86.6	84.9	86.9	82.1	89.7	86.9	85.1	90.4
**Attitude towards vaccination**								
When I think/thought about getting vaccinated against COVID‐19 it makes/made me feel anxious (0–10) ($\bar{X}$±SD)	2.8 ± 3.1	2.0 ± 3.0	2.9 ± 3.2*	3.1 ± 3.3	2.8 ± 3.3	2.4 ± 2.8	3.1 ± 3.3	2.2 ± 2.8
**Perception of the efficacy of the COVID‐19 vaccine**								
Flu vaccination also protects against COVID‐19 infection	4.6	7.5	4.2	4.1	5.9	4.1	3.1	7.8
Receiving a dose of the COVID‐19 vaccine protects more than 90% of the vaccinated population from the disease for years to come	13.1	9.4	13.7	16.3	13.3	9.8	14.9	8.8
Vaccination against COVID‐19 reduces the vaccinated person's risk of getting sick	93.3	96.2	92.2	95.1	95.6	89.3	92.0	95.7
Vaccination against COVID‐19 reduces the possibility of infecting other non‐vaccinated people	52.7	50.0	53.2	51.2	55.2**	50.8	47.3	64.9
It is not worth getting vaccinated because we have effective treatments	1.3	1.9	1.2	0.8	0.7	2.5	1.5	0.9
**Reasons for getting vaccinated against COVID‐19**								
Protect myself	94.9	92.5	95.2	95.1	95.6	93.0	93.5	98.3
Protect my family and friends	96.9	96.2	97.0	98.3	97.0	95.0	96.1	99.1
Working in high‐risk areas	91.5	84.9	92.5	92.7	94.8*	86.9	90.0	94.7
Personal health problems	41.2	37.7	41.7	26.4	44.1	53.7**	38.8	47.8
Protect patients	94.1	96.2	93.7	91.8	95.6	94.2	93.8	94.8
Decrease transmission of the virus	95.6	94.3	95.8	97.5	94.8	94.2	96.5	95.0
Responsibility as a health care worker	95.1	96.3	94.9	93.5	96.3	95.0	94.2	96.5
Avoid sick leave due to infection	67.9	59.6	69.1	60.6	69.6	73.5	68.2	68.7
Recommendation from my superiors at work	31.1	25.0	32.1	21.9	34.9	38.3*	32.0	29.8
**Ethical responsibilities regarding vaccination**								
I believe that getting vaccinated against COVID‐19 will have a positive influence on my patients’ acceptance of the vaccine	80.7	81.1	80.7	83.6	80.8	79.0	79.7	82.6
I believe that getting vaccinated against COVID‐19 is an ethical duty	84.6	88.4	84.0	82.1	85.2	86.9	83.9	85.1
I will recommend my patients get vaccinated	94.0	98.0	93.4	95.2	94.8	93.4	92.3	98.2*
**Perception of the safety of the COVID‐19 vaccine**								
I am convinced that the COVID‐19 vaccine is safe	87.2	94.2	86.1	81.3	87.9	91.6	86.0	90.2
COVID‐19 vaccines could lead to common side effects such as pain, hardening of the injection site, fever, headache, muscle pain, malaise	98.4	100	98.2	99.2	98.5	97.5	98.4	98.3
COVID‐19 vaccines could present very rare side effects such as gene modification and infertility	20.5	17.3	21.0	21.1	24.4	15.8	19.3	22.8***
COVID‐19 vaccines could present rare side effects such as facial swelling, muscle weakness, facial paralysis or anaphylaxis.	64.0	60.8	64.5	73.6	60.0	59.1	60.8	69.6*
I prefer to wait for others to get vaccinated against COVID‐19 first	13.8	17.0	13.2	18.0	14.0	10.0	14.3	13.9
COVID‐19 vaccines could be harmful to pregnant women	38.0	41.2	37.5	47.9	32.6	36.5	40.6	33.3
COVID‐19 vaccines could be harmful to people with chronic diseases	12.1	7.7	12.8	16.4	8.1	12.1	15.0***	6.1
**Perception of the severity of COVID‐19 infection**								
COVID‐19 is a major problem and serious enough to warrant the development of a vaccine	98.4	98.1	98.5	99.2	98.5	97.5	98.5	98.2
I think I am vulnerable to getting sick from COVID‐19	55.0	52.8	55.4	50.4	52.9	61.5	51.7	60.9
I would rather get sick than get vaccinated	3.9	5.8	3.6	2.4	5.9	3.3	4.2	3.5
Most cases of COVID‐19 infection do not present severe symptoms	51.1	58.5	49.9	50.0	56.6	45.9	49.2	53.9
Natural immunity after having been infected is better than getting vaccinated against COVID‐19	23.8	21.2	24.3*	32.8	18.4	20.8	27.5	15.7

* *p* < 0.05.

** *p* < 0.01.

*** *p* < 0.001.

### 5.4 Attitude Towards Vaccination

Nurses in the 35–49 age group believed that vaccination against COVID‐19 reduced the risk of infecting other unvaccinated people (*p* < 0.01). Among the reasons for getting vaccinated, working in high‐risk areas stood out for the 35–49 age group (*p* < 0.05), while personal health problems (*p* < 0.01) and recommendations from superiors (*p* < 0.05) stood out for those over 50. Recommending the COVID‐19 vaccine to patients (*p* < 0.05), the belief that vaccination could lead to infrequent side effects such as facial swelling, muscle weakness, facial paralysis and anaphylaxis (*p* < 0.05), in addition to very infrequent side effects such as gene modification or infertility (*p* < 0.001) all scored higher among PC nurses, while the belief that vaccination could be harmful to people with chronic diseases was higher for hospital nurses (*p* < 0.001). More women than men believed that natural immunity from COVID‐19 infection was better than immunity from vaccination (*p* < 0.05) (Table 3).

## 6 Discussion

This study aimed to analyse the perceived threat of COVID‐19, vaccination coverage and associated factors among nurses in Barcelona in 2021 after the start of the vaccination campaign. Perceived threat was higher among female nurses, the 35–49 age group and the hospital setting. Almost all the nurses were vaccinated. The overall perceived threat was 30.7 points in a study conducted on the general population during the first wave when no vaccine was available (Pérez‐Fuentes et al. 2021). In comparison, it was 29.1 points in the present study, carried out during the third and fourth waves, when the vaccination campaign was already underway. The result shows a slight decrease in threat perception between the first and third waves (before and after the commercialization of the vaccines). This was to be expected since the analysis of the aforementioned study showed no significant association between having been vaccinated and perceived threat. This finding may be related to the fear caused by the initially high death rate of becoming infected and giving COVID‐19 to their relatives (Cho et al. 2021) since the vaccines marketed up until then do not prevent infection, although they are effective in reducing more severe disease and mortality (Comité Asesor de Vacunas 2021). Moreover, of all the HCW profiles, nurses have the most contact with patients (Ganslmeier et al. 2021). In addition to nurses’ mistrust in the safety of the vaccine due to potential side effects and their history of mistrust in annual vaccines such as for influenza (Pinatel et al. 2022), the need for annual revaccination might lead them to consider the vaccine ineffective. If vaccination against COVID‐19 were to become annual, it could decrease adherence among nurses, as is the case with vaccination against seasonal influenza, for which coverage is around 40% (Torner et al. 2016).

Although COVID‐19 infection has affected both men and women of all age groups, perceived threat was higher in women than men. This finding coincides with other studies conducted both on the general population and HCWs in Spain, where the difference in means was greater than 2 points. This might be due to the condition of women as caregivers in our society, where caregiving is still a responsibility perceived as feminine and assumed by women by default. This may have had an impact by imposing on women the need to plan risk‐reduction measures for the people under their care (Pérez‐Fuentes et al. 2021). Not in vain, the fear of returning home and potentially infecting family members at the start of the first wave led many nurses to isolate themselves, staying in hotels or rooms assigned by hospitals. Others chose to send their children to the homes of relatives, which caused feelings of hopelessness, fear and anxiety in a paradoxical situation of having to care for others instead of being able to care for their loved ones (Coşkun Şimşek and Günay 2021).

Regarding age, the results were not significant, which is in line with previous studies where perceived threat was not associated with age, although it was slightly higher among the 35–49 age group. This may be because professionals in this age group are more likely to live with a dependent since it has been shown that having children is related to a greater perceived threat due to the fear of them becoming ill (Pérez‐Fuentes et al. 2021).

In terms of the work setting, there were no differences between perceived threat in nurses working at different levels of care. However, a past study showed that PC staff reported greater anxiety associated with perceived threat than hospital staff. This is because PC is the patient's first point of contact with the health system. As a result, PC workers may have faced greater uncertainty at the start of the pandemic (Londoño‐Ramírez et al. 2021).

Vaccination coverage of nurses was 86.6%, which is very similar to other studies conducted around the same time in the US and Germany, where vaccination coverage of health professionals was 85% and 83.5%, respectively (Kozak and Nienhaus 2021). A study of 91 countries demonstrated that those living in a context with a high human development index were more accepting of the vaccine (Askarian et al. 2022). This might explain the high vaccination coverage in the present study, which exceeded the 70% considered necessary to achieve herd immunity (Esperanza Gómez and Ruiz‐Santa‐Quiteria, n.d.). The under‐35 age group had the lowest vaccination rate. This finding coincides with research from other settings, where young people and women were the groups that showed the greatest hesitancy to be vaccinated against COVID‐19, both among HCWs and the general population (Kozak and Nienhaus 2021). On the one hand, the proliferation of erroneous or contradictory information on digital platforms could have affected young people's vaccination perceptions and decisions, making them more hesitant to get vaccinated (Dzieciolowska et al. 2021). In addition, young people who perceive their health as good or excellent tend to show greater hesitancy towards vaccination, possibly due to the belief that they do not need additional protection (Khan et al. 2021). On the other hand, there is evidence that indicates that women are more accepting than men of non‐pharmaceutical protection measures, such as hand washing, the use of hand sanitizer, gloves and masks, etc., which may reduce the perceived need to be vaccinated (Li et al. 2021). Despite all this, in this study, vaccination coverage was higher among women, although they had higher scores for anxiety related to thinking about getting vaccinated against COVID‐19. This could be due to the constant changes in the safety protocols and guidelines (Franklin and Gkiouleka 2021), which might have lowered the credibility of the measures proposed to mitigate the pandemic.

Regarding the nurses’ attitude towards vaccination, it is associated with trust in the efficacy and safety of the vaccine, perceived personal risk, accessibility and the responsibility to protect others (Ganslmeier et al. 2021). All these factors were observed in the present study. Nurses’ trust in the efficacy and safety of the vaccine was around 90%. All nurses had the opportunity to get vaccinated and the main motivation to do so was to protect others (family and friends). In a study conducted in the US shortly before this one (January 2021), just when the first vaccines were being commercialised there, it was shown that perceived safety in the general population was 66.4% (Kricorian et al. 2022). There are no studies from around the same time that analyse the perceived safety and efficacy in HCWs. The most common safety concerns were the rapid development of the vaccine and side effects (Ganslmeier et al. 2021). In this study, although the perceived efficacy and safety were high in general, it should be noted that 20.5% believed that the vaccine could present very rare side effects such as gene modification and infertility. This phenomenon has been observed in other studies, where some women showed concern about the safety of the vaccine in terms of potential effects on their reproductive health and breastfeeding (Khan et al. 2021).

### 6.1 Limitations of the Work

This study has some limitations. During the pandemic, an online questionnaire was used as it was the best available option and had also been used by international organisations (Eurofound 2022) as it was impossible to conduct in‐person surveys. Since the questionnaire was anonymous, it was not possible to ensure that each person only responded once. The response rate could not be accurately estimated as the number of workers who read the email invitation to participate and the number of nurses who received the link to the questionnaire via snowballing are unknown. Even so, the number of participants exceeded the sample size initially calculated. As usual in online questionnaires, the response rate was low (Díaz de Rada 2012), even though two reminders were sent inviting HCWs to participate to increase the response rate (Sánchez Fernández et al. 2009). It is possible that some nurses with a temporary contract do not frequently check their institutional email inbox. To improve the sample's representativeness, nurses with any type of employment contract were included, as well as hospital and PC nurses. The questionnaire was created based on a literature review, since there was no validated questionnaire on attitudes and practices related to COVID‐19, in addition to the Questionnaire on the Perceived Threat of COVID‐19, which is validated in the Spanish population. Finally, the knowledge about COVID‐19 vaccination has much improved during the current years. It therefore contributes relatively new knowledge in this field.

### 6.2 Implications and Recommendations for Further Research

Based on our results, we first propose improving the management of perceived threat. To do so, meetings could have been held during the pandemic to discuss updates to the protocols and the reasoning behind any changes. Training might also have been offered on how to manage the physical and mental overload of the pandemic and how to improve the consultation of scientific resources to obtain accurate, evidence‐based information.

Nursing education should be adapted to include specialised training on pandemic preparedness, focusing on critical areas such as crisis communication, vaccination science and public health strategies. This could be done through simulations since providing students with realistic outbreak scenarios allows them to practice decision‐making and response strategies in a controlled environment. These updates to the curriculum are essential to ensure nurses are well‐equipped to address the challenges posed by health emergencies.

Secondly, to promote vaccination coverage among young people (the least vaccinated group) and clarify potential doubts regarding safety, strategies should be proposed to promote vaccination. Public confidence must be increased in this sense. This can be achieved through communication and education, such as vaccination reminders and debunking fake news—social networks are a major channel among young people and useful for sharing scientific information. Formal collaborations between governments, healthcare providers and social media companies can ensure the dissemination of accurate health information, combat misinformation and amplify the scope of public health messages through trusted channels.

In addition, policies should support ongoing professional development for nurses and other healthcare professionals. Continuous education initiatives will enable the workforce to stay updated on the latest evidence‐based practices and technologies, ensuring preparedness for future health emergencies.

Lastly, longitudinal studies are needed to monitor perceived threat when COVID‐19 becomes an endemic disease, as well as its association with vaccination coverage if vaccination is done annually, evaluating the scope of dissemination for vaccination and giving greater visibility to epidemiological data related to infection and vaccination.

## 7 Conclusions

One year into the pandemic and with the vaccination campaign already underway, based on the morbidity and mortality of COVID‐19 it was to be expected that perceived threat among nurses would be significant and very similar to that of the general population before the campaign. Women presented a higher perceived threat than men. The attitude of the nurses towards vaccination was good since they trusted the efficacy and safety of the vaccine despite believing some fake news.

## Author Contributions

Marina Moreno‐Martínez: conceptualization, data curation, formal analysis, methodology, writing – original draft; Maria Feijoo‐Cid: conceptualization, methodology, writing – review and editing; Josep Maria Manresa‐Domínguez: Formal analysis, writing – review; Antonia Arreciado Marañón: conceptualization, writing – review; Esperanza Zuriguel‐Pérez: resources, writing – review; Sandra Ponce‐Ruíz: resources, writing – review; Ramón Sebastián Torrente‐Jimenez: writing – review; Maria Isabel Fernández‐Cano: conceptualization, methodology, writing – review and editing.

## Funding

The authors have nothing to report.

## Disclosure

Statistics: The authors have checked to make sure that our submission conforms as applicable to the Journal's statistical guidelines. There is a statistician on the author team: Josep Maria Manresa‐Dominguez. The author(s) affirm that the methods used in the data analyses are suitably applied to their data within their study design and context and the statistical findings have been implemented and interpreted correctly.

## Conflicts of Interest

The authors declare no conflicts of interest.

## Supporting information


**Table S1:** Survey questions used in the study.

## Data Availability

The data that support the findings of this study will be openly available in CORA.RDR.
