# Supplementary material for: Perceived Threat of COVID‐19 and Vaccination Coverage Among Nurses: A Multicentre Cross‐Sectional Study
Source: Nurs Open. 2026 Mar 9;13(3):e70469. doi: 10.1002/nop2.70469 (PMC12968891; doi:10.1002/nop2.70469)
Supplement: Supplementary file 1 — Table S1: Survey questions used in the study. [file NOP2-13-e70469-s001.docx]

Supplementary Table 1: Survey questions used in the study

| AREA | QUESTION | SOURCE |
| --- | --- | --- |
| Sociodemographic and employment data (sex, age, dependents, work center(s), type of contract, and work experience) | 1-10 | Ad hoc |
| Experience with COVID-19 (front-line work, COVID-19 diagnostic tests performed and reason for doing so, positive PCR test or antibody test, compliance with quarantine, and friend/family member affected by COVID-19) | 11-17 | Ad hoc |
| Questionnaire on the Perceived Threat of COVID-19 | 18 | Perez-Fuentes MC, et al. 2020 |
| Vaccination coverage | 19 | Based on a report from the Nurses Professional Association of Spain, Oct. 2020 |
| Attitude towards COVID-19 vaccination (motivation for vaccination, perceived vaccine efficacy and safety, ethical responsibility to get vaccinated, and perceived severity of the problem) | 20-26 | Based on a report from the Nurses Professional Association of Spain, Oct. 2020 |
